# Geographic Structuring of the *Plasmodium falciparum* Sarco(endo)plasmic Reticulum Ca2+ ATPase (PfSERCA) Gene Diversity

**DOI:** 10.1371/journal.pone.0009424

**Published:** 2010-02-25

**Authors:** Ronan Jambou, Axel Martinelli, João Pinto, Simonetta Gribaldo, Eric Legrand, Makhtar Niang, Nimol Kim, Lim Pharath, Béatrice Volnay, Marie Therese Ekala, Christiane Bouchier, Thierry Fandeur, Pedro Berzosa, Agustin Benito, Isabel Dinis Ferreira, Cynthia Ferreira, Pedro Paulo Vieira, Maria das Graças Alecrim, Odile Mercereau-Puijalon, Pedro Cravo

**Affiliations:** 1 Institut Pasteur de Dakar, BP 220, Dakar, Senegal; 2 Institut Pasteur, Paris, France; 3 Centro de Malaria e Outras Doenças Tropicais/IHMT/UNL, UEI Biologia Molecular, UEI Malária, Lisbon, Portugal; 4 Institut Pasteur de Guyane Française, Cayenne, French Guiana; 5 Institut Pasteur du Cambodge, Phnom Penh, Cambodia; 6 Centro Nacional de Medicina Tropical, Instituto de Salud Carlos III, Madrid, Spain; 7 Fundacao de Medicina Tropical do Amazonas, Manaus, Brazil; Texas A&M University, United States of America

## Abstract

Artemisinin, a thapsigargin-like sesquiterpene has been shown to inhibit the *Plasmodium falciparum* sarco/endoplasmic reticulum calcium-ATPase PfSERCA. To collect baseline *pfserca* sequence information before field deployment of Artemisinin-based Combination therapies that may select mutant parasites, we conducted a sequence analysis of 100 isolates from multiple sites in Africa, Asia and South America. Coding sequence diversity was large, with 29 mutated codons, including 32 SNPs (average of one SNP/115 bp), of which 19 were novel mutations. Most SNP detected in this study were clustered within a region in the cytosolic head of the protein. The PfSERCA functional domains were very well conserved, with non synonymous mutations located outside the functional domains, except for the S769N mutation associated in French Guiana with elevated IC_50_ for artemether. The S769N mutation is located close to the hinge of the headpiece, which in other species modulates calcium affinity and in consequence efficacy of inhibitors, possibly linking calcium homeostasis to drug resistance. Genetic diversity was highest in Senegal, Brazil and French Guiana, and few mutations were identified in Asia. Population genetic analysis was conducted for a partial fragment of the gene encompassing nucleotide coordinates 87-2862 (unambiguous sequence available for 96 isolates). This supported a geographic clustering, with a separation between Old and New World samples and one dominant ancestral haplotype. Genetic drift alone cannot explain the observed polymorphism, suggesting that other evolutionary mechanisms are operating. One possible contributor could be the frequency of haemoglobinopathies that are associated with calcium dysregulation in the erythrocyte.

## Introduction

*Plasmodium falciparum* malaria claims close to one million deaths every year, mainly in sub-Saharan African children. To face increasing anti-malarial treatment failure, many countries have adopted artemisinin-based combination therapy (ACT) [1]. However there are concerns about emerging resistance to artemisinin derivatives, with *in vitro* drug resistance to artemether reported in French Guiana and northern Brazil [2]–[3] and high *in vivo* tolerance evidenced by delayed parasite clearance emerging in Cambodia [4]–[8]. The mechanism of action of artemisinin derivatives and the molecular basis of artemisinin resistance and/or tolerance are poorly understood. It has been recently proposed that artemisinins inhibit PfSERCA, the *P. falciparum* Sarco(endo)plasmic reticulum Ca2+ATPase [9]–[10], and thus possibly act by perturbing calcium homeostasis of the intracellular parasite.

SERCA, the calcium pump of sarcoplasmic reticulum responsible for refilling calcium in the ER stores is critically important for cellular homeostasis and calcium signalling functions [11]–[13]. Unlike vertebrates that possess three *serca* genes [14], *P. falciparum* has a single SERCA, originally described as PfATPase6 [15]. Other Apicomplexan parasites such as *Toxoplasma gondii* and *Cryptosporidium parvum* also have a single copy *serca* gene [16]. SERCA proteins are evolutionary well conserved and are composed of three cytoplasmic domains (A, actuator; N, nucleotide binding; P, phosphorylation), ten transmembrane (M1–M10) helices constituting the transmembrane gate and small lumenal loops. Together the A, N and P domains participate in regulating calcium binding and release into the ER lumen. The whole molecule undergoes complex conformational changes during activation, involving a cooperative binding of two Ca2^+^ and phosphorylation of the cytosolic head of the molecule leading to conformational transitions opening the transmembrane channel [11], [17]–[18]. Thapisgargin, a highly cell-penetrant sesquiterpene-lactone that once inside cells potently and specifically inhibits mammalian SERCA, binds to a cavity at the interface of the M3, M5 and M7 helices [19]–[20]. Importantly, specific inhibition of SERCA by thapsigargin kills cancer cells by provoking the apoptotic cell death pathway [21].

The sesquiterpene-lactone structure of thapsigargin, which is a highly specific SERCA inhibitor, is structurally reminiscent of artemisinin. Interestingly, both thapsigargin and artemisinin were shown to inhibit the ATP hydrolysis activity of the PfSERCA produced in the heterologous *Xenopus laevis* oocyte expression system [9]. Consistent with the notion of PfSERCA inhibition, thapsigargin antagonized the parasiticidal activity by artemisinins [9]. In line with this, artemisinin-susceptibility of the PfSERCA protein produced in the *Xenopus laevis* oocyst heterologous expression system was abolished by introduction of a single L263E point mutation [22]. Remarkably, a few years after the introduction of artemether combination therapy in French Guiana, a S769N PfSERCA polymorphism was observed in several patient isolates presenting with a markedly increased 50% Inhibitory Concentration (IC_50_) for arthemether and hence were classified as *in vitro*-resistant [2]–[3]. However, this S769N polymorphism was also reported in one African isolate, which was susceptible to dihydroartemisinin *in vitro* [23]. It is worth noting that artemisinin-resistant mutants of the murine malaria parasite *P. chabaudi chabaudi* [24] or the related Apicomplexan parasite *T. gondii* had an unaltered *serca* gene sequence though calcium homeostasis was altered in these artemisinin-resistant *T. gondii* parasites [25]. Thus, although the relationship between *Pfserca* polymorphism and *P. falciparum* susceptibility to artemisinin remains to be confirmed, it is conceivable that artemisinin-resistance involves species-specific mechanisms, as shown for e.g. chloroquine [26].

Insights into the relative impact of *pfserca* polymorphisms on the biological function of the protein on the one hand and its susceptibility to artemisinins on the other hand may be gained from an analysis of field polymorphisms. Apart from the above mentioned novel S769N mutation observed in French Guiana [2]–[3] and Africa [23], a number of other *pfserca* polymorphisms have been reported in laboratory lines [27] and in field isolates from Africa [2], [28]–[29], although their relationship with artemisinin susceptibility remains unknown. An intriguing aspect is the relatively large *pfserca* polymorphisms that they show relatively large sequence diversity in comparison to other so-called house keeping genes, such as the lactate dehydrogenase locus studied in the same lines [27]. To further explore *Pfserca* sequence diversity and to look for possible causes and geographical structuring, we sequenced a large panel of field isolates from various malaria-endemic geographic areas before widespread deployment of ACT. Our data show clear evidence for geographic structuring and indicate that as yet unknown evolutionary mechanisms are contributing to the large field polymorphism of this locus in some areas.

## Results

### *Pfserca* Polymorphism in the Field

We sequenced *pfserca* in 100 isolates collected from South America, Asia and Africa (Table 1). Full length coding sequence was determined for 56 isolates, and partial (positions 87-2862 of the gene sequence) was obtained for 40 isolates. Overall, 29 codons carried one or more SNP. Codons 538, 569 and 987 harboured two mutations. A total of 32 distinct SNPs were observed, of which 19 were non synonymous (NS) and 13 synonymous mutations, resulting in an average of one SNP/115 bp (Table 2). 19 mutations had not been described previously. Haplotype reconstruction allowed grouping samples into 28 distinct haplotypes/alleles (Table 3).

**Table 1:** Details of samples for which *pfserca* sequence was studied.

Continent	Collection area	No samples	Year of collection	Sequenced*	Reference of sample collection
AFRICA	Equatorial Guinea	9	2005–2006	Partial	unpublished
	Senegal	16	2003	Full-length	Jambou et al., 2005
AMERICA	Brazil (Amazonas)	10	2003–2006	Partial	unpublished
	Brazil (Pará)	13	2005	partial	Ferreira et al., 2007
	French Guiana	20	2003	Full-length	Jambou et al., 2005
ASIA	Cambodia	24	2005	Full-length	Jambou et al., 2005
	Thailand	8	2002	partial	Lopes et al., 2002

*gene region sequenced:

full-length refers to entire coding sequence, corresponding nucleotide 1-3687 (cordon 1-1228), partial refers to region encompassing nucleotide coordinates 87-2862 in the Pfserca 3D7 reference sequence (PFA0310c).

**Table 2:** List of the *pfserca* mutations detected.

Nucleotide position	Codon No	Wild-type codon	Mutant Codon	Aminoacid (NS: bold; Syn: *italics*)	Country of origin
**110**	37*	AGA	AAA	**R – K**	Brazil
**266**	89	ATA	ACA	**I – T**	Thailand
**421**	141*	TGG	GGG	**W – V**	Senegal
**1204**	402	TTA	GTA	**L – V**	Brazil French Guiana Senegal
**1262**	421**	GGA	GAA	**G – E**	French Guiana
**1291**	431**	AGA	AAA	***E – K***	Senegal
**1323**	441**	TAT	TAC	*Y – Y*	French Guiana
**1329**	443**	GAT	GAC	*D – D*	French Guiana
**1395**	465	AAT	AAC	*N – N*	French Guiana
**1612**	538	AGT	GGT	***S – R***	French Guiana
**1614**	538	AGT	AGC	*S – S*	French Guiana
**1616**	539	ACC	ATC	**T – I**	French Guiana
**1671**	557	TCT	TCA	*S – S*	Equatorial Guinea
**1706**	569**	AAT	AGT	***N – S***	Equatorial Guinea
**1707**	569**	AAT	AAA	**N – K**	Equatorial Guinea
**1710**	570**	ACA	ACC	*T – T*	French Guiana
**1721**	574**	ACA	CCA	**Q – P**	French Guiana
**1861**	621**	GCT	TCT	**A – S**	French Guiana
**1868**	623**	GCA	GAA	**A – E**	Senegal
**1888**	630**	GCT	TCT	**A – S**	Brazil
**1916**	639**	GGC	GAC	**G – D**	Brazil
**1927**	643**	GAA	CAA	**E – Q**	Equatorial Guinea
**2049**	683	AAT	AAG	**N – K**	Senegal
**2306**	769	AGT	AAT	**S – N**	French Guiana
**2656**	885*	ATT	ATA	*I – I*	Senegal
**2694**	898*	ATA	AAT	*I – I*	Brazil French Guiana Equatorial Guinea Senegal
**2877**	959	ATT	ATA	*I – I*	Cambodia
**2960**	987	ATT	CTT	**I – T**	Senegal
**2962**	987	ATT	ATA	*I – I*	Cambodia
**2974**	998	AGT	AGC	*S – S*	Senegal
**3090**	1030*	AAG	AAA	*K – K*	Cambodia
**3093**	1031*	TGC	TGT	*C – C*	Cambodia, Senegal

Legend: NS: non synonymous. Syn: synonymous. All nucleotide, codon and corresponding amino acid positions in the table are adjusted to correspond to positions in the reference 3D7 sequence (accession n. PFA0310c) (http://www.plasmodb.org/plasmo/). Catalytic domains (*): cation atpase_N AA 3 – 81 and atpase_C, AA 1031–1215, E1_E2 atpase AA 99 – 348/AA 358–364, hydrolase AA 790–935; *Plasmodium* specific area (**): AA 407–486, 569–671, 852–860.

**Table 3:** Estimates of genetic diversity per sample and neutrality tests.

	*S (η_s_)*	*h*	*H_d_*	*K*	*π*	*D*	D*	F*
French Guiana	9 (5)	12	0.889	2.084	0.00075	−0.614^ns^	−1.203^ ns^	−1.197^ ns^
Pará	4 (1)	3	0.564	1.538	0.00055	0.657^ ns^	0.335^ ns^	0.474^ ns^
Amazonas	2 (0)	3	0.689	0.822	0.00030	0.526^ ns^	1.026^ ns^	1.010^ ns^
Senegal	6 (2)	9	0.908	1.800	0.00065	−0.016^ ns^	−0.047^ ns^	−0.044^ ns^
Eq. Guinea	5 (4)	6	0.889	1.444	0.00052	−0.910^ ns^	−1.239^ ns^	−1.291^ ns^
Thailand	2 (1)	3	0.464	0.679	0.00024	−0.448^ ns^	−0.149^ ns^	−0.238^ ns^
Cambodia	0 (0)	1	n.a.	n.a.	n.a.	n.a.	n.a.	n.a.
America	13 (5)	15	0.818	1.998	0.00072	−1.032^ ns^	−0.943^ ns^	−1.147^ ns^
Africa	10 (6)	13	0.910	1.727	0.00062	−1.150 ^ns^	−1.654^ ns^	−1.751 ^ns^
Asia	2 (1)	3	0.140	0.209	0.00008	−1.241^ ns^	−0.714^ ns^	−0.995^ ns^
All samples	21 (10)	28	0.742	1.500	0.00054	−1.855*	−2.501*	−2.698*

Legend: The extent of *pfserca* gene sequence analysed here correspond to nucleotide coordinates 87-2862 of the PFA0310c *pfserca* sequence of the reference clone 3D7 (Plasmodium genome database PlasmoDB; http://www.plasmodb.org/plasmo/). *N*: sample size. *S*: number of segregating sites, of which the number of singletons *(η_s_)* is presented in parenthesis. *h*: number of haplotypes. *H_d_*: haplotype diversity (*i.e.* expected heterozygosity based on haplotype frequency). *K*: average number of nucleotide differences between two sequences. *π*: average number of nucleotide differences between two sequences, per site. *D*: Tajima's (1989) values for *D*-test. D* and F* are Fu & Li's (1993) values for the respective neutrality tests. ^ns^: not significant. *P<0.05. n.a.: not applicable (monomorphic).

**Figure 1:**
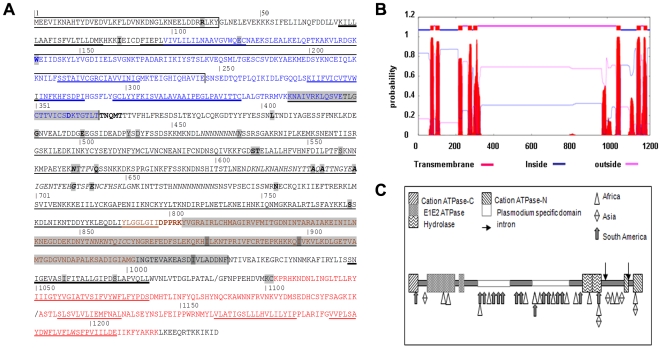
Predicted domains and localization of the mutations in Pf-SERCA protein sequence. A) Amino-Acid sequence of Pf-SERCA The functional domains of the protein are located according to the alignment with the rabbit ATPase: A (boxed), P (box-grey) and N (underlined), *Plasmodium*-specific domains are shown in italic. Hinge between P and N domains are bolded (TNQMT and DPPRK). Introns are located in the gene after L1019 and K1119 (noted by the sign/). Transmembrane domains are bold-underlined. Mutations are boxed in grey (non-synonymous mutation in bold). Three catalytic domains are predicted by PROSITE: cation atpase_N 3 – 81 and ATPase_C 1031–1215 (red), E1_E2 atpase 99 – 348/358-364 (blue), hydrolase 790–935 (braun). The position of the two introns is indicated with a/. B) Prediction of transmembrane domain by HMM (www.PROSITE.org) Ten transmembrane domains were predicted using TMpred with “in to out” (i-o) or “out to in”(o-i) orientation: M1 AA67-85 (o-i), M2 AA94-116 (i-o), M3 AA216-233 (o-i), M4 AA271-290 (i-o), M5 AA298-325 (o-i), M6 AA979-1005 (i-o), M7 AA1053-1073 (o-i), M8 AA1124-1140 (i-o), M9 AA1158-1175 (o-I), M10 AA1183-1206 (i-o). C) Schematic representation of the catalytic domains of Pf-SERCA and localization of the mutations. Mutations are given symbols by continent of origin of the isolates (non synonymous mutations depicted with filled arrows, synonymous mutations depicted with open arrows).

The predicted domain structure of PfSERCA based on alignment of th*e* reference PfSERCA protein sequence with other sequences selected across the phylogenetic tree and the identified functional and structural domains [30]–[35] (detailed in supplementary Figures S1 and S2 with Supporting Information File #1) is presented in Figure 1 A and B. As previously noted by other authors [27], [29], PfSERCA has the largest sequence of all those deposited in NCBI as it contains specific inserts absent from all other SERCAs. These inserts are mainly located in the N domain facing the cytoplasmic side of the ER. The location of the various SNPs observed in this panel of isolates is shown in Figure 1C. Three SNPs were located within the A domain (codons 37, 89 and 141), all three were NS mutations. Twenty one SNPs were located within the N-domain, 11 of which within the *Plasmodium*-specific sequences (with 7 mutated codons –codons 569, 570, 621, 623, 630, 639 and 643 within a *falciparum/reichenowi*-specific insert). Of the 21 SNPs affecting the N domain, only six were synonymous. In contrast, seven of eight SNPs located in within the P-domain were synonymous mutations, including two SNPs within a transmembrane domain. 3Five SNPs (R37K, W141G, S538S, S538R, T539I) were located in the vicinity of residues essential for the catalytic activity of the protein. No mutation was identified in the amino acids described as of major importance for the SERCA function in animal species (ATP, phosphate and Ca++ binding, reviewed in [18]).

### PfSERCA Polymorphism and Its Relationship with *In Vitro* Susceptibility to Artemisinins

In the set of 100 patient isolates studied here, 60 had their *in vitro* susceptibility profile assessed for an artemisinin derivative, either artemether or artesunate [2]. Each of the isolates presenting an IC50 greater than 20 nM had a distinct haplotype, but some commonalities were observed. In French Guiana three isolates had an IC_50_ for artemether greater than 100 nM; their haplotype was [L402V N569S S769N I898I], [E431K 769N I898I], [L402V S538S Q574P S769N I898I]. The haplotype of two isolates from French Guiana with an IC_50_ for artemether in the 50–100 nM range was [S538S S769N I898I] and [S569S I898I]. Five isolates from French Guiana, which presented IC_50_ for artemether between 20 and 50 nM had the following haplotype [S769N I898I], [L402V S539R Q574P S769N I898I], [L402V S538R I898I], [I898I], [D443D S769N I898I]. In Senegal there were three isolates with an IC_50_ for artesunate between 20 and 45 nM, their haplotypes were [E431K A623E], [E431K] and [L402V A623E]. Of these mutations, only S769N, which was associated with a high level of *in vitro* artemether resistance (IC_50_ above 30 nM) in French Guiana. S769N is located outside the *falciparum*-specific insert, in a position close to the C-terminal hinge between the N- and P- domains.

### Genetic Diversity and Geographic Distribution

A total of 96 *pfserca* sequences were analysed for a fragment of 2785 bp encompassing nucleotides 87-2862. Nine positions corresponding to tri-nucleotide microsatellite repeats (AAT) starting at position 1292 were excluded from further analyses. Thus, we analysed a partial fragment of 2776 bp, which corresponded to nucleotide coordinates 87 – 2862 in the *pfserca* 3D7 reference sequence (accession n. PFA0310c) deposited at the Plasmodium genome database PlasmoDB. Overall, 21 variable sites were detected, of which 10 were singletons (positions 266, 421, 1262, 1329, 1612, 1671, 1707, 1710, 1868 and 1927) and 11 were parsimony informative (positions 110, 1204, 1291, 1614, 1706, 1721, 1888, 1916, 2049, 2306 and 2694). Of the 21 segregating sites, 5 were synonymous changes and 16 were non-synonymous mutations (Table 2).

Parasites from Africa and America presented the highest estimates of *pfserca* haplotype diversity, *H_d_* (Table 3). Africa showed comparable estimates of variability among samples (*i.e.* countries) whereas in South America, the sample of French Guiana was the only showing values of *H_d_* comparable to Africa. The two Brazilian samples had lower haplotype diversity, in particular Amazonas'. A similar trend was observed in terms of nucleotide diversity (Table 3), although in this case the South American sample from Pará presented an estimate of *π* intermediate to those obtained for African samples. Diversity for the *pfserca* gene from Asia was very low. The sample from Cambodia was monomorphic, while the sample from Thailand displayed the lowest estimates of haplotype and nucleotide diversities.

Tajima's [36] and Fu & Li's [37] (Table 3) tests showed no significant departures from neutrality, either within country or continents. Only when all sequences were pooled were significant departures (*P*<0.05) observed in all tests, but this probably results from ascertainment bias due to sampling of a non-randomly mating gene pool. The *d*_N_/*d*_S_ ratios were below one, indicative of purifying selection, except for the sample of Amazonas in which no synonymous mutations were detected (Table 4). However, differences between *d*_N_ and *d*_S_ were non-significant in all samples. For the McDonald-Kreitman test, marginally significant (*P*<0.05) departures from neutrality associated with *NI* values above one (indicative of purifying selection) were detected in the samples of French Guiana and Senegal (Table 4). These tests were not considered significant after applying Bonferroni corrections for multiple tests (adjusted significance level: α' = 0.009).

**Table 4:** *d*_N_ */d*_S_ ratio and McDonald-Kreitman test of neutrality for the *pfserca* gene.

	*d*_N_ */d*_S_ ratio		McDonald-Kreitman test					
			Interspecific fixed nucleotide differences		Intraspecific nucleotide polymorphisms			
	*d*_N_/*d*_S_	*P*-value^1^	*Syn*	*Nsyn*	*Syn*	*Nsyn*	*NI*	*P-value* ^2^
**French Guiana**	0.53	0.511	43	15	3	6	5.7	0.022
**Pará**	0.59	0.707	43	15	1	3	8.6	0.070
**Amazonas**	∞	0.169	43	15	0	2	n.a.	0.077
**Senegal**	0.75	0.824	43	15	1	5	14.3	0.010
**Eq. Guinea**	0.22	0.318	43	15	2	3	4.3	0.136
**Thailand**	0.15	0.415	43	15	1	1	2.9	0.475
**Cambodia**	n.a.	n.a.	43	15	0	0	n.c.	n.c.

Legend *P-*value^1^: two-tailed Z-test based on 1000 bootstraps with an alternative hypothesis of *d*_N_≠*d*_S_. *Syn*: synonymous. *Nsyn*: non-synonymous. *NI*: neutrality index. *P-*value^2^: *P-value* of Fisher's Exact test. ∞: infinite (no synonymous polymorphisms). n.a.: not applicable (no intraspecific synonymous polymorphisms). n.c.: not computed (monomorphic sample).

The extent of *pfserca* gene sequence analysed here correspond to nucleotide coordinates 87-2862 of the PFA0310c *pfserca* sequence of the reference clone 3D7 (Plasmodium genome database PlasmoDB; http://www.plasmodb.org/plasmo/).

Overall, genetic differentiation of *pfserca* (global *F*_ST_ = 0.214, *P*<0.001) reflected the geographic origin of samples. Highly significant *F*_ST_ estimates (*P*<0.001) were obtained for the three pair wise comparisons: America vs. Africa (*F*_ST_ = 0.097), America vs. Asia (*F*_ST_ = 0.088), Africa vs. Asia (*F*_ST_ = 0.117). In Asia, all comparisons involving the monomorphic sample of Cambodia were significant, with the exception of the neighbour sample from Thailand (Table 5). In Africa, no significant genetic differentiation was observed between the two samples of this continent, (*F*_ST_ = 0.066). In contrast, American samples showed high pair wise *F*_ST_ values, particularly in comparisons involving Amazonas, which supports a high *P. falciparum* population subdivision in this area.

**Table 5:** Genetic differentiation between samples, measured by *F*_ST_.

	French Guiana	Pará	Amazonas	Senegal	Equatorial Guinea	Thailand
French Guiana	-					
Pará	**0.213**	-				
Amazonas	**0.308**	0.341	-			
Senegal	**0.179**	0.136	**0.336**	-		
Equato Guinea	0.190	0.077	**0.367**	0.066	-	
Thailand	0.175	0.092	0.412	0.078	−0.019	-
Cambodia	**0.198**	0.231	**0.543**	**0.200**	**0.188**	0.095

Legend: Underlined: *P*<0.05; In bold: significant estimate after Bonferroni correction. The extent of *pfserca* gene sequence analysed here correspond to nucleotide coordinates 87-2862 of the PFA0310c *pfserca* sequence of the reference clone 3D7 (Plasmodium genome database PlasmoDB; http://www.plasmodb.org/plasmo/).

**Figure 2:**
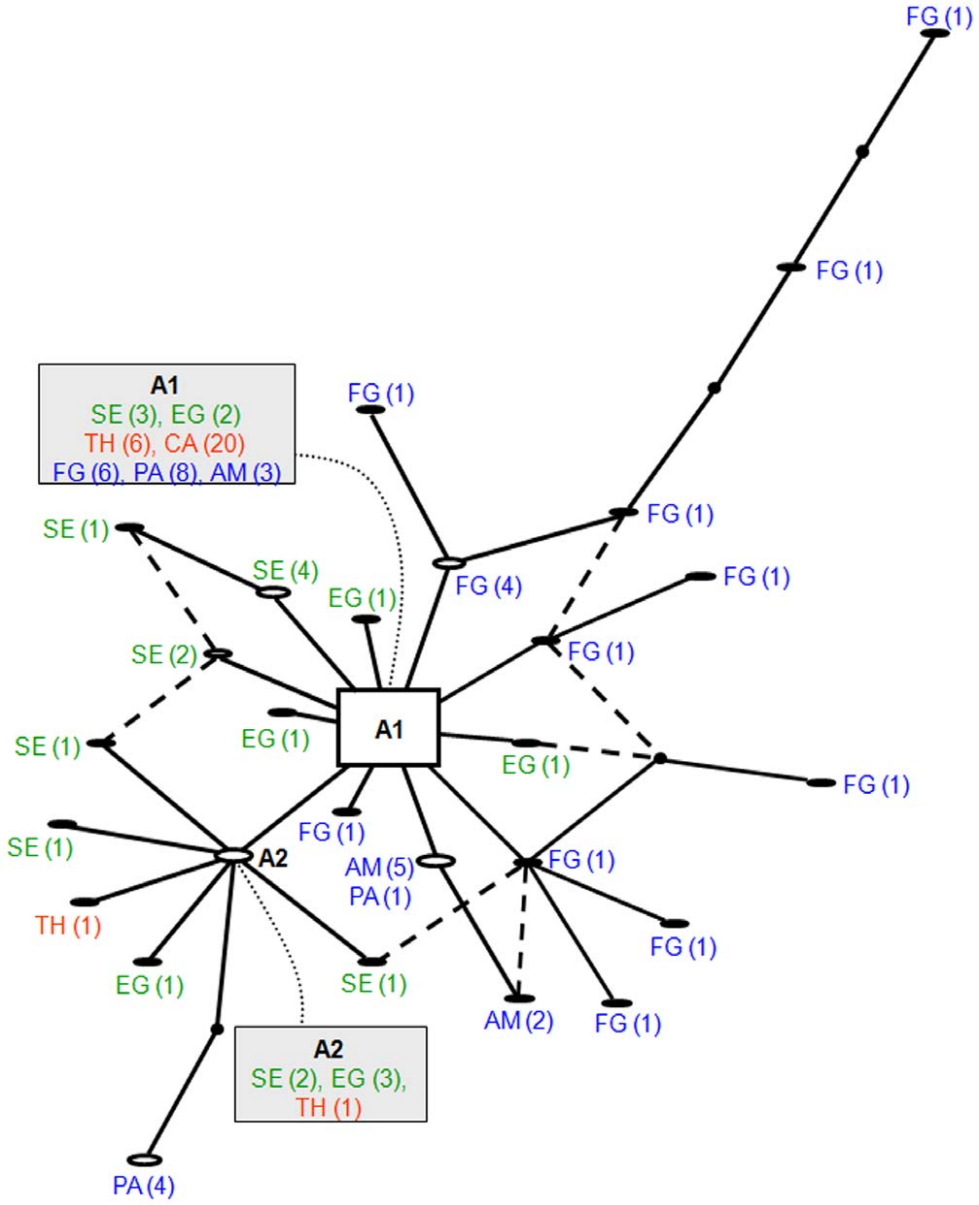
TCS network for the pfserca gene of Plasmodium falciparum. Each distinct haplotype is represented by an oval with size proportional to its frequency (in parenthesis). Mutational steps are represented by lines. Dots connecting lines represent putative haplotypes that were not sampled. Dashed lines refer to reticulations corrected according to Crandall et al. [38]. Colours correspond to each continent sampled: blue - America (AM: Amazonas; FG: French Guiana; PA: Para), green - Africa (EQ: Equatorial Guinea; SE: Senegal), red - Asia (CA: Cambodia; TH: Thailand).

To analyse this geographic subdivision, a genealogical network was produced using the TCS software with correction of the reticulations [38] (Figure 2). The ancestral haplotype (A1) was present in all samples and it was the predominant haplotype in the Asian ones. The second most represented haplotype (A2) was present only in Africa and Asia. All remaining haplotypes were region or country-specific. Samples from French Guiana revealed a complex genealogical pattern derived from A1 and formed by unique branches separated from A1 by up to six mutational steps with low-frequency internal clades.

## Discussion

The large sequence diversity observed in this analysis confirms findings from previous *pfserca* molecular surveys. For instance, in Niger six different mutations were found [28] while Dahlstrom *et al.* [29] found 31 mutations in 302 samples from Zanzibar and three mutations in 39 Tanzanian samples. Similarly Menegon *et al.* [39] found seven mutations in 71 specimens. The synthesis of all reported polymorphisms is provided in Table S1 (with Figure - References S1). The overall degree of *pfserca* polymorphism in our study was one SNP/115 bp and when including all published sequences (some of which being quite partial) this figure was one SNP/55 bp. This somehow puts *pfserca* at odds compared to genes presenting similar GO classification. These figures are indeed much higher than estimated average of one SNP/910 bp in coding regions [40], one common SNP per 842 bp [41], one SNP/780 bp across three fully sequenced genomes and one every 446 bases across certain large genomic regions [42]. Considering that intronic sequences that evolve more rapidly than coding sequences were excluded from our analysis, our SNP rates are if anything going to have been underestimated. Kidgell et al reported that genes located in the central regions of chromosomes (as is *pfserca*) usually present minimal sequence diversity [43]. Interestingly however, as noted in chromosome or genome wide studies [41]–[42] most SNP were rare, with 15 SNPs observed once and only 6 SNPs presenting a>5% frequency in the samples analysed. As a result, in the set of samples studied, a minority of SNPs (3 of 32) were observed in multiple settings, and a similar conclusion can be drawn when analysing the compiled list of SNPs reported to date, with only 14 of 68 mutations observed in multiple settings.

Parasites from Africa and America were the most genetically diverse. Within the African continent samples showed comparable estimates of variability among the different countries sampled. Samples from Asia presented the lowest genetic diversity. These observations are in line with a previous report indicating reduced diversity in Asian *P. falciparum* parasites inferred by typing of 183 SNPs across chromosome 3 [40], but contrast with other studies showing lower genetic diversity in South America than in Asia [44]–[46]. For instance, Mehlotra et al report a much lower *Pfcrt* and *Pfmdr* haplotype diversity in samples from South America, including Brazil and Guyana – which borders French Guiana- compared to parasites from African areas [45], contrasting with our observation of similar, large diversity in Africa and South America for *pfserca*. Neafsey et al studied a large panel of SNPs across the 14 chromosomes in a set of isolates including isolates from Brazil, Senegal and Thailand –three geographic areas sampled here as well- and found that the lowest diversity was in Brazil [46]. In our study, the lowest diversity of the three areas was Thailand. The reason of our discrepancy with the latter studies is unclear but this may be due to differences in technical approach used to study polymorphism or differences in sampling or in the genomic regions analysed, since the degree of genetic differentiation in *P. falciparum* has been shown to vary across the genome. Moreover we did not include the non coding regions in our analysis and did not survey intronic or flanking 5′ or 3′ microsatellite diversity, known to be much larger than coding sequences.

Importantly, genetic differentiation at the *pfserca* gene reflected the geographic origin of samples. When samples were pooled by continent, highly significant (P<0.001) *F*_ST_ estimates were obtained for the three pair wise comparisons (0.088≤*F*_ST_≤0.117). This is in line with numerous studies of chromosomal or mitochondrial genes [40]–[43], [46]. Within continents, the monomorphic sample of Cambodia was not significantly different from the neighbour sample from Thailand, and virtually no population substructure was observed in Africa. In contrast, samples from the American continent showed significantly high pair wise *F*_ST_ values, particularly in comparisons involving the sample from Amazonas. This suggests a much higher *P. falciparum* population substructure is detected by this gene in South America, a conclusion in agreement with the notion of a higher *P. falciparum* population differentiation in regions of lower malaria transmission [44]. Interestingly, estimates of genetic variation in America were greatly influenced by the French Guiana cohort, in which diversity was comparable to Africa. This may be due to the particular micro-epidemiological scenario in French Guiana, with patchy foci, each with a small number of inhabitants having easy access to antimalarial drugs, a situation that favours outgrowth of variant parasites. This was further confirmed by a genealogy displaying multiple tip haplotypes suggesting a local recent expansion of *pfserca* diversity. The peculiar epidemiology of the French Guiana area was also outlined by a previous study of the mitochondrial *cytb* gene, which displayed a particularly elevated polymorphism with specific SNPs, some reaching very high frequency [47].

In the TCS genealogical network, the most frequent haplotype was represented in samples from all continents surveyed and it is also likely to be the ancestral haplotype of *pfserca*. No clear overrepresentation of samples from a particular continent occurred within this haplotype and all internal haplotypes originating from the ancestor were distributed equally between South America and Africa. The network revealed a high number of low frequency external clades, which may be indicative that the gene is evolving rapidly. The particular reasons why this happens are difficult to disentangle from epidemiological or gene-specific function scenarios. *Pfserca* is the only serca gene of *Plasmodium*, making it highly specialized and unique for the parasite's calcium homeostasis. Interestingly, it has been recently suggested that highly specialized genes in the *Plasmodium* genome are more likely to undergo accelerated selective processes [48]–[49]. However, neither Tajima's nor Fu & Li's tests provided evidence of departures from neutrality and calculations of the McDonald-Kreitman test identified only marginally significant departures in French Guiana (*P* = 0.022) and in Senegal (*P* = 0.010). These were associated with *NI* values >1 indicative of purifying selection. Although the P-values were not considered significant after Bonferroni corrections for multiple tests, it was interesting to notice that these were the two countries where decreased *in vitro* susceptibility of *P. falciparum* to an artemisinin derivative was reported at the time of sample collection [2]. Purifying selection acts to prevent divergence of structure and function by selecting against extreme values of a trait, in contrast to directional selection, where a single phenotype is selected and therefore allele frequency shifts in one direction. The latter type of natural selection is more likely to occur on drug resistant determinants but probably does not apply for the present set of samples collected before substantial artemisinin pressure was in place in all the areas surveyed. Furthermore, the two most frequent SNPs were synonymous and their frequency varied greatly with the area/continent surveyed. The I898I mutation reached very high local frequency in Asia (0.78 in Cambodia, 0.85 in Thailand), but also in other areas such as Brazil (local frequency 0.85) or Equatorial Guinea (local frequency 0.50), while they were not detected at all in Senegal or French Guiana. Although we cannot exclude that such synonymous mutations may not be phenotypically silent as some synonymous *mdr* mutations were shown to influence the rate of translation, protein folding and substrate specificity [50], we tentatively conclude that it is likely that neutral evolution operating on possible multiple colonising events is currently shaping polymorphism at the *pfserca* gene of *P. falciparum* at this stage in history i.e. before massive worldwide deployment of artemisinin derivatives. Multiple colonisation events by ancestral parasites spreading out of Africa to the other continents have been suggested by the analysis of mitochondrial DNA [51].

The most obvious and statistically significant association regarding artemisinin susceptibility was that of the 769N mutation with an increased IC_50_ for artemether [2]. This was only observed in the set of samples from French Guiana. The mutation is located in the vicinity of the predicted hinge domain of the protein, which influences binding of the calcium ion. As the inhibitory effect of thapsigargin depends on the engagement of the first calcium ion in the binding site [17], [30], [52], [53], it is possible that the 769N mutation decreases the capacity of artemisinin to impair this process. This mutation was not detected in Asia and in the vast majority of African isolates studied so far [28]–[29], except in one dihydroartemisinin-susceptible isolate whose *in vitro* susceptibility to artemether remains unknown [23]. The marked geographical clustering of isolates from Asia, Africa and America observed here may account for the particular artemether resistance/*pfserca* mutations relationship found in America, especially as high levels of *in vitro* resistance to artemether have only been observed in America so far and as the genetic background of parasites from South America differs from Asia or Africa [40]–[44], [46]. The association of 769N with artemether susceptibility and its relationship to the *in vitro* susceptibility to dihydroartemisinin certainly needs to be further studied. To ascertain the functional role of this mutation, biochemical studies with the mutant enzyme are needed along with genetic exchange studies in *P. falciparum* parasites. It is however possible that some mutations such as 769N have a heavy fitness cost, as parasites harbouring the mutation could not be propagated *in vitro* under standard culture conditions.

An important question for future studies is whether or not *pfserca* genetic characteristics change over time and in particular under the pressure by artemisinin derivatives delivered as combination therapies. In this regard, a recent survey of haplotype diversity for two chloroquine resistance candidate genes, *pfcrt* and *pfmdr1*, provides important clues for interpreting genetic variation of drug resistance candidate genes in light of drug pressure. This study showed that overall genetic variation in the *pfcrt* gene was lower than in the *pfmdr1* gene because chloroquine has exerted a stronger selective pressure on *pfcrt* favouring the occurrence of selective sweeps around the locus, resulting in reduced genetic diversity with time [45]. Because *pfmdr1* is a putative modulator of responses to artemisinin combination therapies as well as of chloroquine, future patterns of *pfmdr1* variation may help understand the specific selective pressures of the newly implemented combination therapy policy. The present data will serve as a useful snapshot to infer how *pfserca* polymorphisms is affected by drug pressure and whether some SNPs/haplotypes are selected by artemisinin combination therapies.

The significance of the large *pfserca* polymorphism and the consequences of the mutations described here on the protein's function and regulation are unclear. As indicated above, the dominant allele in all settings was the 3D7-type supposedly coding for a wild type sequence. In Cambodia, all mutations were synonymous, and likewise all but one sample from Thailand had only synonymous SNPs. This suggests substantial functional constraints. In line with the bioinformatic modelling [35] alignment of the PfSERCA sequence with other proteins outlined high conservation of all functional domains and transmembrane helices of the protein. PfSERCA is the largest SERCA described so far due to the presence of large *falciparum*-specific inserts most of which map to a region of the extra-membranous head that in other SERCAs is associated with closing the calcium channel. This suggests that these inserts could influence the channels properties by altering the conformational changes associated with calcium transport, and/or cope with specific requirements associated with intracellular calcium homeostasis in the human erythrocyte or hepatocyte host cell. As artemisinin was shown to inhibit *T. gondii* SERCA and trigger calcium release [54], the link between PfSERCA and calcium homeostasis deserves detailed investigation. This could furthermore offer new insights on factors possibly contributing to the high level of polymorphism of PfSERCA. Interestingly, the areas where the highest PfSERCA diversity was observed in this work and in previous studies are those with an elevated prevalence of haemoglobinopathies such as HbS, namely African populations [55] and South American countries with a substantial population of African origin (e.g. Belize, French Guiana and north Brazil). Sickle cell anaemia and beta-thalassaemia are associated with an elevation of total red cell calcium content [56]–[59]. This abnormally high cytosolic calcium content could be a selective force for mutant PfSERCA as an enhanced activity of this pump is needed in such environments to restore physiological intracellular calcium stores [60]–[61]. This possibility is currently under investigation.

## Materials and Methods

For more details see also Materials and Methods S1.

### Ethics Statement

This program received ethical clearance from all the National Ethical Committees of the countries involved in the study (NEC of Senegal, Cambodia, Brazil, Thailand, and the CCPRB of French Guiana). All the patients enrolled (or their family) gave a written informed consent for the protocol.

### Sample Collection

Blood samples were obtained from consenting patients attending dispensaries with uncomplicated *P. falciparum* malaria (Table 1). The following isolates were used as characteristic haplotypes: F126 (GU456693), F128 (GU456694), F119 (GU456695), F91 (GU456696), F94 (GU456697), F64 (GU456698), F139 (GU456699), F132 (GU456700), F122 (GU456701), F108 (GU456702), F41 (GU456703), AM54 (GU456704), AM46 (GU456705), BR67 (GU456706), 8A (GU456707), 66A (GU456708), 22A (GU456709), 22G (GU456710), 24G (GU456711), 7A (GU456712), 58A (GU456713), 80A (GU456714), 9A (GU456715), EQG66 (GU456716), EQG41 (GU456717), EQG72 (GU456718), EQG28 (GU456719), RC17 (GU456720).

### Sequencing of Field Isolates

Parasite DNA was extracted from red blood cells by standard phenol/chloroform method. Primers used were synthesized based on the published sequence of *Pfatp6* (Genbank, AN AJ532679). Samples from Thailand, Equatorial Guinea, Brazil 5amazonas and Parà) were sequenced as described [62]. Samples from French Guiana, Senegal and Cambodia were amplified using the following primers pairs (numbering refers to indicates the position of the first nucleotide of the primer in the PFA0310c sequence): 20 5′ATGCTCATACATACGATGTTGAG/1607 5′ AATATGAACAGTGATCCTCAGAC; 1268 5′ TTGAAGCTTTAACGGATGATGGA/3039 5′ TACCTAGTGCTGTTGCTGGTAA; 3207 5′ AATCCACCAGAACATGACGTAAT/3457 5′AATATGAACAGTGATCCTCAGAC; 3335 5′ TAGGCAAGCACCTTATCTTTATC/3952 5′ TTTTCTTGGTTCTTTGCTCTTCC. PCR reactions were done in 50 µL in the presence of 2.5 mM MgCl2, 2.5 U AmpliTaq Gold (Applied Biosystems). An initial denaturation at 94°C for 9 min, was followed by 38 cycles at 94°C for 1 min, 60°C for 2.5 min (for primers 20/1607 and 1268/3039, and 3 min at 56°C for primers 3207/3457 and 3335/3952) 72°C for 2 min followed by one cycle were the last elongation was for 10 min. PCR products were sequenced on both strands using internal primers essentially as described [47]. Potential sequence ambiguities were resolved either by re-sequencing or through close inspection of the corresponding chromatogram, using PhredPhrap (www.phrap.org).

### Bioinformatic Analysis

Sequences have been aligned using CLUSTALX software (version1.81) [63] and BOXSHADE (www.pasteur.fr). Functional areas were predicted using PROSITE. Prediction of the PfSERCA domains was performed by alignment with a set of other eukaryotic serca (*P. yoelii*, *P. gallinaceum*, *P. knowlesi*, *P. berghei*, *P. reichenowi*, *P. vivax*, *Oryctolagus cuniculus*, *Homo sapiens*, *Mus musculus*, *Rattus norvegicus*, *Caenorhabditis elegans*, *Leishmania donovani*, *Trypanosoma cruzi*). Helix prediction was performed using TMpred software (www.expasy.ch).

### Phylogenetic Analysis of the Coding Sequence

Sequence polymorphism analysis and neutrality tests were performed using the software DnaSP 4.50.3 [64]. Neutrality was tested using Tajima's [36] and Fu & Li's [37] tests. Departures from selective neutrality at the protein level were assessed by the *d*_N_ */d*_S_ ratio and the McDonald-Kreitman test. For between-species comparisons, a sequence of the *ATPase6* gene from the closely related *P. reichenowi* (GenBank n° AB122148.1) was used. For *d*_N_ */d*_S_ ratios, significance was determined by a two-tailed Z-test available in MEGA 4.0.2 [65]. Fisher's exact tests on 2×2 contingency tables were used for McDonald-Kreitman tests, as implemented in DnaSP [66].

Levels of genetic differentiation were measured by the fixation index *F*_ST_, based on nucleotide frequency differences [64]. To assess if *F*_ST_ values significantly differed from zero, permutation tests were performed based on the *K*_ST_* estimator of pair wise nucleotide differences [64]. Calculations were performed with DnaSP. A genealogical network was produced using the TCS software [67], with correction of the reticulations following Crandall and Templeton [38].

Whenever multiple tests were performed, the nominal significance level (α = 0.05) was adjusted by Bonferroni corrections to avoid type I error (i.e. false rejection).

## Supporting Information

Figure S1 Mapping of the three main domains in Pf-SERCA Legend Three major motifs were detected using PROSITE: A) pfam00122.11 (E1-E2 ATPase) (residues 107-357 of PfSERCA); B) pfam00689.11 (Cation_ATPase_C) (residues 1032-1215 of PfSERCA), and C) pfam00702.11 (Hydrolase) (residues 790-931 of PfSERCA). Domains are mapped with sequences characteristic of the functions (indicated by accession number). In red conserved amino acid of the motifs.
(0.47 MB PPT)

Figure S2 Alignment of Pf-SERCA with other known SERCA to point out amino acids of interest in the falciparum protein. Alignment of the SERCA protein sequences from five Plasmodium species with ten sequences selected across the phylogenetic tree. PfSERCA has the largest sequence of all those deposited in NCBI. The global SERCA structure is easily identifiable, with a cytosolic head composed of three domains homologous to the rabbit A, P, and N domains. Note that the PfSERCA and the P.reichenowi SERCA sequence contain specific inserts absent from all other SERCAs. These inserts, mainly located in the N domain located on the cytoplasmic side of the ER contain no identifiable motif. The region was predicted to be part of the extra-membranous head responsible for closing the Ca++ channel during the E1-E2 conformational change. The integral membrane domain of the protein is composed of ten helix domains, in line with other SERCAs. The two calcium pockets are completely conserved with only a shift in the position in the sequence due to a larger head. The key P-phosphorylation residue [1] was predicted to be D357, in line with the analysis of Nagamune et al [2]. In other SERCAs, phosphorylation of this aspartate in the presence of calcium induces a major conformational E1-E2 transition in the enzyme, associated with the release of the cation into the luminal side of the membrane (reviewed in [3]). This movement involves two well conserved hinges [4]–[6] that are also present in PfSERCA: i) at the interface of N and A domains (motif 601DPPRK in rabbit SERCA, corresponding to motif 798DPPRK in PfSERCA), and ii) between N and P domains (308TNQM in rabbit, 405TNDI in PfSERCA). Along the same line, C674 recently identified as a key residue in the NO-induced glutathione regulation of the rabbit SERCA, was identified as C887 in the PfSERCA sequence [7]–[8]. The thapsigargin binding site involving (F256-I765-Y837) in the human SERCA can putatively be located in the PfSERCA (F264-I976-Y1048, Figure S2), and is juxtaposed to the putative artemisinin site (L263, I272, F273, I1041) as proposed by in silico docking after deleting falciparum-specific inserts [9]. Three major domains are mapped: A domain (in blue), N domain (in green), P domain (in red). Conserved amino acids are bolded. Three numbering of amino acids are shown, P.falciparum, rabbit and human.
(0.42 MB XLS)

Materials and Methods S1 (0.04 MB DOC)

Table S1 (0.14 MB PDF)

References S1 (0.00 MB RTF)
