# Supplementary figures and images for: Geographic Structuring of the Plasmodium falciparum Sarco(endo)plasmic Reticulum Ca2+ ATPase (PfSERCA) Gene Diversity
Source: PLoS One. 2010 Feb 25;5(2):e9424. doi: 10.1371/journal.pone.0009424 (PMC2828472; doi:10.1371/journal.pone.0009424)

## Slide 1
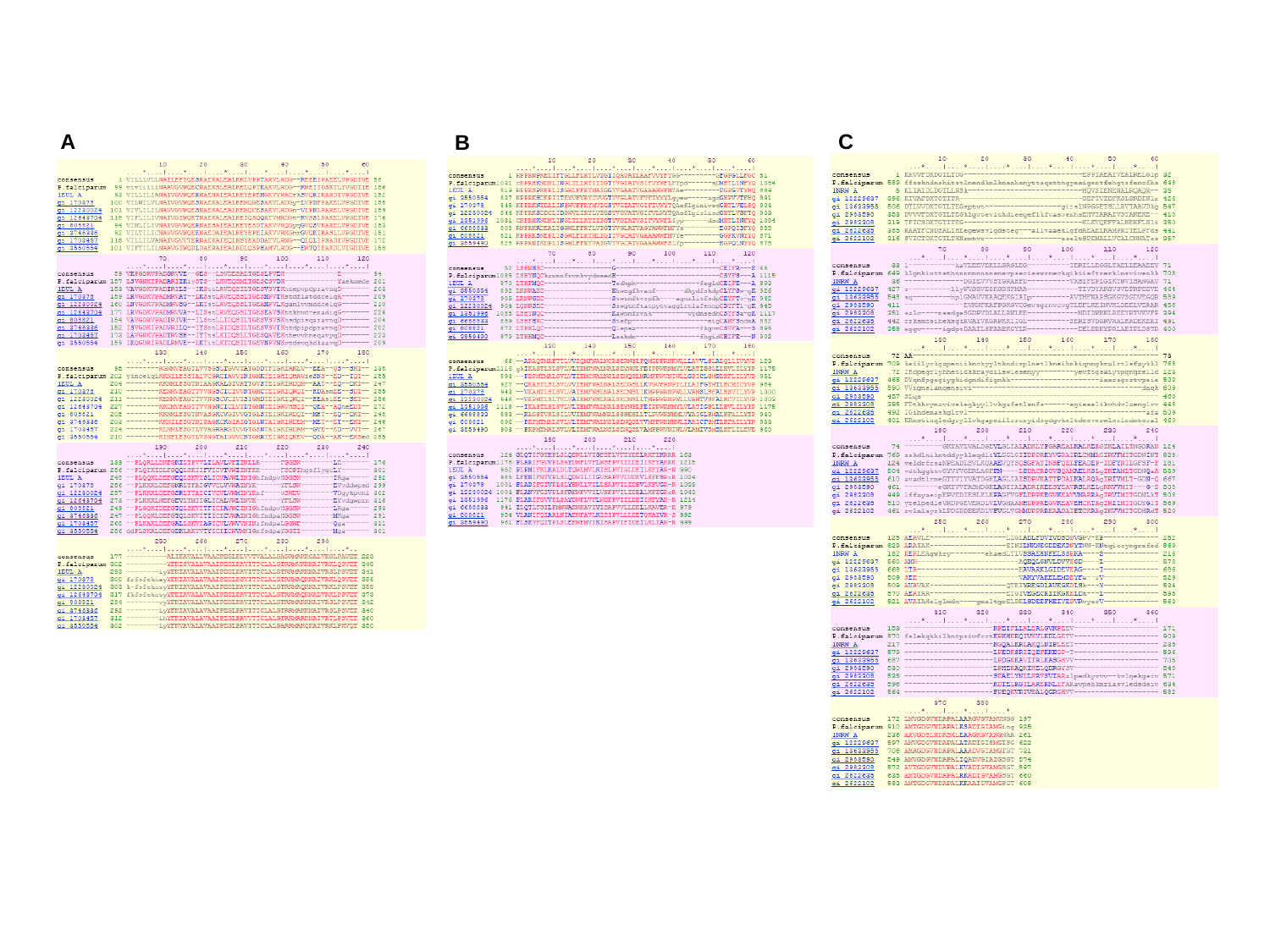

A
C
B

Supplement: Figure S1 — Mapping of the three main domains in Pf-SERCA Legend Three major motifs were detected using PROSITE: A) pfam00122.11 (E1-E2 ATPase) (residues 107-357 of PfSERCA); B) pfam00689.11 (Cation_ATPase_C) (residues 1032-1215 of PfSERCA), and C) pfam00702.11 (Hydrolase) (residues 790-931 of PfSERCA). Domains are mapped with sequences characteristic of the functions (indicated by accession number). In red conserved amino acid of the motifs. (0.47 MB PPT) [file pone.0009424.s001.ppt]
