# Supplementary material for: Geographic Structuring of the Plasmodium falciparum Sarco(endo)plasmic Reticulum Ca2+ ATPase (PfSERCA) Gene Diversity
Source: PLoS One. 2010 Feb 25;5(2):e9424. doi: 10.1371/journal.pone.0009424 (PMC2828472; doi:10.1371/journal.pone.0009424)
Supplement: Materials and Methods S1 — (0.04 MB DOC) [file pone.0009424.s003.doc]

**Material and methods, Online supplement**

**Genetic analysis**

Neutrality at the genomic level, was analysed using Tajima’s (1989) and Fu & Li’s (1993. For Fu & Li’s (1993) tests, negative values of estimates are obtained when there is an excess of mutations in the external branches of the gene genealogy (*i.e.* singletons). This suggests that this region may be affected by purifying or negative selection, or that an advantageous allele may have recently become fixed in the population (Fu & Li, 1993). Similarly, negative Tajima's (1989) *D* values result from an excess of low frequency polymorphisms, suggesting population size expansion and/or purifying selection. Positive Tajima's *D* values signify low levels of both low and high frequency polymorphisms, indicating a decrease in population size and/or balancing selection.

Departures from selective neutrality at the protein level were assessed by the *d*_N_*/d*_S_ ratio and the McDonald-Kreitman test. The *d*_N_*/d*_S_ ratio is the quotient between the number of non-synonymous substitutions per non-synonymous site (*d*_N_) and the number of synonymous substitutions per synonymous site (*d*_S_). If *d*_N_*/d*_S_ ≈1, the sequence is neutrally evolving, since the likelihood that a non-synonymous will go to fixation is the same as that for a synonymous mutation. Values of *d*_N_*/d*_S_ >1 indicate that the sequence is evolving under positive selection whereas *d*_N_*/d*_S_ < 1 indicates purifying selection. To assess whether *d*_N_ significantly differed from *d*_S_, one-tailed Z-tests were conducted by a bootstrap resampling method (500 replicates) with a null hypothesis of *d*_N_*=d*_S_ and alternative hypothesis *d*_N_<*d*_S_ or *d*_N_>*d*_S_. Calculations were done in MEGA 3.1 (Kumar *et al.*, 2004). In the McDonald-Kreitman test, the ratio of non-synonymous to synonymous polymorphisms within a species (Pn/Ps) is compared to the ratio of non-synonymous to synonymous fixed differences between species (Dn/Ds). If polymorphism and divergence are driven only by mutation and genetic drift (*i.e.* under neutrality), the ratio of the number of fixations to polymorphisms should be the same for both non- synonymous and synonymous mutations. The neutrality index *NI* [computed as *NI*= (Pn/Ps)/(Dn/Ds)] measures the extent to which levels of polymorphism departure from expectations under the neutral model. Under neutrality *NI*=1. *NI*>1 is interpreted as an excess of within-species polymorphic variation due to purifying selection whereas *NI*<1 reflects an excess of variation between species due to positive selection.

Levels of genetic differentiation were measured by the fixation index *F*_ST_, based on nucleotide frequency differences (Hudson et al., 1992a). To assess if *F*_ST_ values significantly differed from zero, permutation tests were performed based on the *K*_st_* estimator of pairwise nucleotide differences (Hudson et al. 1992b). Calculations were performed with DnaSP. A neighbour-joining phylogenetic tree was constructed based on the pairwise *F*_ST_ estimates, using MEGA 3.1. A genealogical network was produced for the *ATPase6* gene of *P. falciparum*, using the TCS software (Templeton et al., 1992).
