# Supplementary material for: Geographic Structuring of the Plasmodium falciparum Sarco(endo)plasmic Reticulum Ca2+ ATPase (PfSERCA) Gene Diversity
Source: PLoS One. 2010 Feb 25;5(2):e9424. doi: 10.1371/journal.pone.0009424 (PMC2828472; doi:10.1371/journal.pone.0009424)
Supplement: Table S1 — (0.14 MB PDF) [file pone.0009424.s004.pdf]

Supplementary Table . List of the mutations observed in *pfserca*

Codon	nucleotide position mutation	wild-type codon	mutant codon	Amino Acid	Isolate	Country of origin of the isolate	Reference
037*	110 G>A	AGA	AAA	R→K	7G8	Brazil[#]	Tanabe 2004<br>Ferreira 2008
089	266 T>C	ATA	ACA	I→T	Mad20<br>T9/94	Papua New Guinea<br>Thailand<br>Vietnam	Tanabe 2004<br>Price 2004<br>Bertaux 2009
141*	421 T>G	TGG	GGG	W→G		Senegal[#]	this work
229	685 G>A	GTA	ATA	V→I		Zanzibar	Dahlström 2008
243	727 C>T	CAT	TAT	H→Y		Angola<br>Zanzibar	Menegon 2008<br>Dahlström 2008
252	756 T>C	GAT	GAC	*D→D*		Zanzibar	Dahlström 2008
402**	1204 T>G	TTA	GTA	L→V	T9/96	Senegal[#], Brazil[#]<br>Thailand<br>Senegal, Ghana, Nigeria, Kenya, Cameroon, Congo Dem, Niger	Tanabe 2004<br>Menegon 2008
421**	1233 G>A	GGA	GAA	G→E		French Guiana[#]	this work
431**	1262 G>A<br>?1291	GAA	AAA	E→K		Senegal[#]<br>Senegal, Angola, Ghana, Cote d'Ivoire, Nigeria, Kenya, Congo Dem, Madagascar, Niger, China<br>Zanzibar, Tanzania, Gambia, Liberia, Malawi Sudan, Uganda	Menegon 2008<br>Dahlström 2008
438**	1313 C>A	GCT	GAT	A→D	Mad20	Papua New Guinea	Tanabe 2004
441**	1323 T>C	TAT	TAC	*Y→Y*		French Guiana[#]	this work
443**	1329 C>T	GAC	GAT	*D→D*		French Guiana[#]	this work
460	1380 T>C			*N→N*		Vietnam	Bertaux 2009
463	1388 A>G<br>1387-1392			N→S<br>del		Vietnam	Bertaux 2009
465**	1395 T>C<br>1395 A>G	AAT	AAC<br>AGC	*N→N*<br>N→S		French Guiana[#]<br>Vietnam	this work<br>Bertaux 2009
466	1397 G>A	AGT	AAT	S→N	HB3	Honduras	Tanabe 2004
537	C>A	GAC	GAT	*D→D*		Niger	Ibrahim 2009
538	1612 A>C<br>1614 T>C	AGT	CGT<br>AGC	S→R<br>*S→S*		French Guiana[#]	this work
539	1616 C>T	ACC	ATC	T→I		French Guiana[#]	this work
557	1671 T>A	TCT	TCA	*S→S*		Equatorial Guinea[#]	this work
561	A>T	AAA	AAT	K→N		Niger	Ibrahim 2009
569**	1706 A>G<br>1706 A>T<br>1707 T>A	AAT	AGT<br><br>AAA	N→S<br>N→I<br>N→K		French Guiana#<br>Zanzibar<br>Papua New Guinea,	this work<br>Dahlström 2008

						Gambia, Zanzibar,<br>Malawi, Tanzania<br>Sudan,<br>Niger	Ibrahim 2009
570**	1710 A>C	ACA	ACC	*T→T*		French Guiana[#]	this work
574**	1721 A>C	CAA	CCA	Q→P		French Guiana[#]	this work
585	*1754 G>A*	GGT	GAT	G→D		Zanzibar	Dahlström 2008
602	1805 C>T	ACA	ATA	T→I		Zanzibar	Dahlström 2008
610	1828 T>A	TTA	ATA	L→I		Zanzibar	Dahlström 2008
611	1833 G>T	AAG	AAT	K→N		Zanzibar	Dahlström 2008
612	1834 A>T	AAT	TAT	N→Y		Zanzibar	Dahlström 2008
621**	1861 G>T<br>1862 C>A	GCT	TCT<br>GAT	A→S<br>A→D		French Guiana[#]<br>Zanzibar	this work<br>Dahlström 2008
623**	1867 G>A<br>1868 C>A	GCA	ACA<br>GAA	A→T<br>A→E		Zanzibar<br>Senegal[#], Senegal	Dahlström 2008<br>Menegon 2008
630**	1888 G>T	GCT	TCT	A→S		Brazil[#]<br>Zanzibar, Tanzania<br>Angola<br>Niger	this work<br>Dahlström 2008<br>Menegon 2008<br>Ibrahim 2009
632	1895 G>A	GGA	GAA	G→E		Zanzibar	Dahlström 2008
635	1903 A>G<br>1904 C>A	ACA	GCA<br>AAA	T→A<br>T→K		Zanzibar	Dahlström 2008
637	1910 A>G	GAG	GGG	E→G		Zanzibar	Dahlström 2008
639**	1916 G>A	GGC	GAC	G→D	7G8	Brazil<br>Brazil<br>Niger<br>Zanzibar	Tanabe 2004;<br>Ferreira 2008<br>Ibrahim 2009<br>Dahlström 2008
641	1921 A>G	AGT	GGT	S→G		Zanzibar	Dahlström 2008
643**	1927 G>C	GAA	CAA	E→Q		Equatorial Guinea[#]<br>Zanzibar	this work<br>Dahlström 2008
644	1931 A>T	AAT	ATT	N→I		Zanzibar	Dahlström 2008
649	1945 A>G	AAA	GAA	K→E		Zanzibar	Dahlström 2008
683	2049 T>G	AAT	AAG	N→K		Senegal[#]<br>Senegal<br>Cambodia<br>Vietnam	this work<br>Menegon 2008<br>Dahlström 2008<br>Bertaux 2009
723	2167 A>G	ATA	GTA	I→V		Zanzibar	Dahlström 2008
747	2239 C>T<br>2240 A>G	CAT	TAT<br>CGT	H→Y<br>H→R		Zanzibar<br>Zanzibar	Dahlström 2008
756	2268 A>G	AGA	AGG	*R→R*		Vanuatu	Dahlström 2008
758	2274 A>G	TTA	TTG	*L→L*		Zanzibar	Dahlström 2008
769	2306 G>A	AGT	AAT	S→N		French Guiana[#]<br>?	Jambou, 2005<br>Cojean 2006
771	2311 A>G	AAA	GAA	K→E		Angola, Ghana,Niger	Menegon 2008
776	2328 G>T	AAG	AAT	K→N		China	Menegon 2008
783	2347 A>G	AAA	GAA	K→E		Zanzibar	Dahlström 2008
801	2403 T>C	CGT	CGC	*R→R*		Zanzibar	Dahlström 2008
809	2425 A>G	AGA	GGA	R→G		Zanzibar	Dahlström 2008

844	2530-2	GGA	del	G→-	HB3	Honduras	Tanabe 2004
885*	2656 T>A	ATT	ATA	*I→I*		Senegal[#]	this work
898*	2694 T>A	ATT	ATA	*I→I*		Brazil[#]<br>Cambodia[#]<br>Brazil[#]<br>Vietnam	this work<br>this work<br>Ferreira 2007<br>Bertaux 2009
959	2877 T>A	ATT	ATA	*I→I*		Cambodia[#]	this work
987	2960 A>C<br>2962 T>A	ATT	CTT<br>ATA	I→T<br>*I→I*		Senegal[#]<br>Cambodia[#]	this work
998	2974 T>C	AGT	AGC	*S→S*		Senegal[#]	this work
1030	3090 G>A	AAG	AAA	*K→K*		Cambodia[#]	this work
1031*	3093 C>T	TGC	TGT	C→C		Senegal[#,] Cambodia[#]<br>Vietnam	this work<br>Bertaux 2009

Legend:
**Plasmodium specific area : AA 407-486, 569-671, 852-860
[#] this work

references

1. Tanabe K, Sakihama N, Hattori T, Ranford-Cartwright L, Goldman I, et al (2004) Genetic distance in housekeeping genes between Plasmodium falciparum and Plasmodium reichenowi and within P. falciparum. J Mol Evol 59: 687-94.
2. Ferreira ID, Lopes D, Martinelli A, Ferreira C, do Rosário VE, Cravo P. In vitro assessment of artesunate, artemether and amodiaquine susceptibility and molecular analysis of putative resistance-associated mutations of Plasmodium falciparum from São Tomé and Príncipe. Trop Med Int Health. 2007 Mar;12(3):353-62.
3. Price RN, Uhlemann AC, Brockman A, McGready R, Ashley E, Phaipun L, Patel R, Laing K, Looareesuwan S, White NJ, Nosten F, Krishna S. Mefloquine resistance in Plasmodium falciparum and increased pfmdr1 gene copy number. Lancet. 2004 Jul 31-Aug 6;364(9432):438-47.PMID: 15288742
4. Bertaux L, Quang le H, Sinou V, Thanh NX, Parzy D. New PfATP6 mutations found in Plasmodium falciparum isolates from Vietnam. Antimicrob Agents Chemother. 2009 Oct;53(10):4570-1.
5. Dahlström S, Veiga MI, Ferreira P, Mårtensson A, Kaneko A, et al (2008) Diversity of the sarco/endoplasmic reticulum Ca(2+)-ATPase orthologue of Plasmodium falciparum (PfATP6). Infect Genet Evol 8: 340-5.
6. Menegon M, Sannella AR, Majori G, Severini C. Detection of novel point mutations in the Plasmodium falciparum ATPase6 candidate gene for resistance to artemisinins. Parasitol Int. 2008 Jun;57(2):233-5.
7. Ibrahim ML, Khim N, Adam HH, Ariey F, Duchemin JB (2009) Polymorphism of PfATPase in Niger: detection of three new point mutations. Malar J 8:28.
8. Jambou R, Legrand E, Niang M, Khim N, Lim P, et al (2005) Resistance of Plasmodium falciparum field isolates to in-vitro artemether and point mutations of the SERCA-type PfATPase6. Lancet 366: 1960-1963
9. Cojean S, Hubert V, Le Bras J, Durand R (2006) Resistance to dihydroartemisinin. Emerg Infect Dis 12: 1798-9.
