# Supplementary material for: Geographic Structuring of the Plasmodium falciparum Sarco(endo)plasmic Reticulum Ca2+ ATPase (PfSERCA) Gene Diversity
Source: PLoS One. 2010 Feb 25;5(2):e9424. doi: 10.1371/journal.pone.0009424 (PMC2828472; doi:10.1371/journal.pone.0009424)
Supplement: References S1 — (0.00 MB RTF) [file pone.0009424.s005.rtf]

References S1: Supplementary references

1.	Ma H, Lewis D, Xu C, Inesi G, Toyoshima C (2005) Functional and structural roles of critical amino acids within the"N", "P", and "A" domains of the Ca2+ ATPase (SERCA) headpiece. Biochemistry 44: 8090-100
2.	Nagamune K, Sibley LD (2006) Comparative genomic and phylogenetic analyses of calcium ATPases and calcium-regulated proteins in the apicomplexa. Mol Biol Evol 23: 1613-27.
3.	Toyoshima C (2009) How Ca(2+)-ATPase pumps ions across the sarcoplasmic reticulum membrane.  Biochim Biophys Acta 1793: 941-6
4.	Inesi G, Lewis D, Ma H, Prasad A, Toyoshima C (2006) Concerted conformational effects of Ca2+ and ATP are required for activation of sequential reactions in the Ca2+ ATPase (SERCA) catalytic cycle. Biochemistry 45: 13769-78.
5.	Toyoshima C, Nomura H (2002) Structural changes in the calcium pump accompanying the dissociation of calcium. Nature 418: 605-11.
6.	Montigny C, Picard M, Lenoir G, Gauron C, Toyoshima C, et al (2007) Inhibitors bound to Ca(2+)-free sarcoplasmic reticulum Ca(2+)-ATPase lock its transmembrane region but not necessarily its cytosolic region, revealing the flexibility of the loops connecting transmembrane and cytosolic domains. Biochemistry 46: 15162-74.
7.	Adachi T, Weisbrod RM, Pimentel DR, Ying J, Sharov VS, et al (2004) S-Glutathiolation by peroxynitrite activates SERCA during arterial relaxation by nitric oxide. Nat Med 10: 1200-7
8.	Cohen RA, Adachi T (2006) Nitric-Oxide-Induced Vasodilatation: Regulation by Physiologic S-Glutathiolation and Pathologic Oxidation of the Sarcoplasmic Endoplasmic Reticulum Calcium ATPase. Trends Cardiovasc Med 16: 109–114
9.	Jung M, Kim H, Nam KY, No KT (2005) Three-dimensional structure of Plasmodium falciparum  Ca2+-ATPase(PfATP6) and docking of artemisinin derivatives to PfATP6. Bioorg Med Chem Lett 15: 2994-2997. 
